# Gene expression in breastmilk cells is associated with maternal and infant characteristics

**DOI:** 10.1038/srep12933

**Published:** 2015-08-10

**Authors:** Alecia-Jane Twigger, Anna R. Hepworth, Ching Tat Lai, Ellen Chetwynd, Alison M. Stuebe, Pilar Blancafort, Peter E. Hartmann, Donna T. Geddes, Foteini Kakulas

**Affiliations:** 1 School of Chemistry and Biochemistry, Faculty of Science, The University of Western Australia, 35 Stirling Highway, Crawley WA 6009, Australia; 2 Department of Obstetrics and Gynecology, Division of Maternal-Fetal Medicine, School of Medicine, University of North Carolina, 3010 Old Clinic Building, CB 7615, Chapel Hill, NC 27599, USA; 3 Department of Pharmacology, School of Medicine, University of North Carolina, 120 Mason Farm Road, Chapel Hill, NC 27599, USA; 4 Cancer Epigenetics group, the Harry Perkins Institute of Medical Research, and School of Anatomy, Physiology and human Biology, The University of Western Australia, 35 Stirling Highway, Crawley WA 6009, Australia

## Abstract

Breastmilk is a rich source of cells with a heterogeneous composition comprising early-stage stem cells, progenitors and more differentiated cells. The gene expression profiles of these cells and their associations with characteristics of the breastfeeding mother and infant are poorly understood. This study investigated factors associated with the cellular dynamics of breastmilk and explored variations amongst women. Genes representing different breastmilk cell populations including mammary epithelial and myoepithelial cells, progenitors, and multi-lineage stem cells showed great variation in expression. Stem cell markers ESRRB and CK5, myoepithelial marker CK14, and lactocyte marker α-lactalbumin were amongst the genes most highly expressed across all samples tested. Genes exerting similar functions, such as either stem cell regulation or milk production, were found to be closely associated. Infant gestational age at delivery and changes in maternal bra cup size between pre-pregnancy and postpartum lactation were associated with expression of genes controlling stemness as well as milk synthesis. Additional correlations were found between genes and dyad characteristics, which may explain abnormalities related to low breastmilk supply or preterm birth. Our findings highlight the heterogeneity of breastmilk cell content and its changes associated with characteristics of the breastfeeding dyad that may reflect changing infant needs.

Human milk (breastmilk) is a complex fluid consisting of a number of diverse components, which are biologically optimised for the human infant^1^. Amongst these are biochemical factors that provide nutrition, immunological support and developmental programming, and which change dynamically both within and between women^2^,^3^,^4^. For example, the lipid content of breastmilk is known to change both during and after breastfeeding and pumping^5^,^6^. Preterm birth influences breastmilk lipid, carbohydrate and energy contents^7^. In addition, protein content decreases over the course of lactation in both term and preterm milk^5^,^8^. Other factors associated with differing breastmilk biochemical composition include parity, maternal body mass index (BMI) and bra size, and infant sex^9^,^10^. Cells are not a negligible component of breastmilk, yet most studies have focused on factors influencing changes in biochemical components. Very little is known about breastmilk cells, variation in gene expression between mothers, and characteristics of the mother/infant dyad that may influence breastmilk cell content^11^. Current knowledge is limited to cellular changes due to feeding/milk removal^6^ and the responses of breastmilk leukocytes to mother and/or infant infections during the course of lactation^12^.

Breastmilk contains a heterogeneous mix of cells including epithelial cells and leukocytes. Leukocytes are the most widely studied cell type in breastmilk due to their protective properties and their known ability to infiltrate the infant’s tissues^12^,^13^,^14^. However, leukocytes constitute only a minority of cells in mature human milk when both the breastfeeding mother and infant are healthy^11^,^12^. On the other hand, epithelial cells are thought to be the most dominant cell type in human milk, and their properties and functions have not been intensively studied^11^.

Breastmilk epithelial cells consist of the two main types of cells, luminal and myoepithelial cells. Luminal cells express epithelial cell adhesion molecule (EPCAM)^11^ whereas myoepithelial cells express smooth muscle actin (SMA) and cytokeratin 14 (CK14)^11^. Luminal epithelial cells are made up of small populations of ductal non-secretory epithelial cells which express cytokeratin 19 (CK19) and alveolar cells (lactocytes) that express cytokeratin 18 (CK18) and synthesize and secrete milk, and are thus positive for milk proteins such as α-lactalbumin (α-LA)^15^ and β-casein^15^. Mammary stem-like cells positive for the markers α6 integrin (CD49f) and cytokeratin 5 (CK5) have also been identified in breastmilk and have been proposed to act as precursors to both luminal and myoepithelial cell types^16^,^17^. Earlier reports have demonstrated that epithelial cells isolated from freshly expressed breastmilk were able to expand in adherent culture and form colonies of various morphologies that could be maintained through multiple passages^15^,^18^,^19^,^20^,^21^. This suggested for the first time the presence of self-renewing cells in breastmilk^11^. These observations together with previous work by Russo *et al.* on the ultra-structure of lactocytes suggested that breastmilk contains both less differentiated, self-renewing cells and more differentiated milk-secretory cells^22^.

Cregan *et al.* (2007) first reported cells with mammary stem-like properties in breastmilk, expressing ectodermal progenitor markers such as Nestin^23^. The presence of these cells has been confirmed by other investigators^16^,^24^ and was further expanded by the identification of breastmilk stem cells (BSCs)^25^. BSCs not only self-renew in 3D spheroid culture, but also express pluripotency genes including the core transcription factors OCT4, SOX2 and NANOG and downstream targets KLF4, REX1 and GDF3. These breastmilk-derived cells are capable of differentiating into cells from all three germinal layers^25^,^26^. The various levels of gene expression observed within single breastmilk cell samples^25^ confirmed the presence of a cellular hierarchy in breastmilk, from early-stage stem cells to progenitor cells to more differentiated lactocytes and myoepithelial cells^11^,^16^,^23^,^24^,^25^.

The discovery of BSCs with multilineage differentiation potential raised numerous questions as to the fate of these cells in the breastfed infant and their potential use in regenerative medicine^11^,^27^. Recent advances in our laboratory have provided the first evidence that BSCs integrate into tissues of the neonate^13^, potentially providing developmental benefits^11^. This, together with previous observations that BSCs are not tumorigenic^25^ and are naturally transferred from the mother to the infant render these cells excellent candidates for stem cell therapies^11^. However, before such applications can be further explored, it is necessary to establish factors influencing the prevalence of breastmilk stem cells in expressed milk in order to optimize their isolation. Therefore, the aim of this study was to describe the existing variation of breastmilk cell populations between women, and to explore associations of gene expression with dyad characteristics, particularly those that have been previously linked to changes in biochemical components^5^,^7^,^9^,^10^. A secondary aim was to examine genes not previously assessed in breastmilk cells to broaden knowledge of the cell types present in human milk and their potential functional significance.

## Results

### Breastmilk cell gene expression varies amongst women

The demographic characteristics of the study participants (n = 66) are described in Table 1. Mothers were on average 34 years of age (range: 21–44) and typically delivered their infant vaginally (n = 48; 35 female infants; 30 male infants; 1 set of male/female twins) at term (gestational age at delivery: 39.3 weeks; range: 27.1–43.4 weeks). At the time of breastmilk sample collection, infants had an average age of 30.5 weeks (range: 1–177 weeks). The maternal BMI was 23.2 (range: 17.5–37.4). Bra size was used as an indicator of breast volume, as previously described^28^. The average change in bra size from pregnancy to lactation was from a C to a D cup. The infants were mainly first born (n = 40), with a range of 2–4 children from multiparous mothers (n = 26). Breastmilk sample volumes collected were on average 55 mL (range: 6–240 mL) and contained on average total cell counts of 2.77 × 10^5^ cells/mL milk (range: 0.17 × 10^5^–3.75 × 10^6^ cells/mL milk). The majority of cells isolated from freshly expressed breastmilk were viable (mean: 97.9%; range: 85.3–100%) (Table 1).

**Figure 1:**
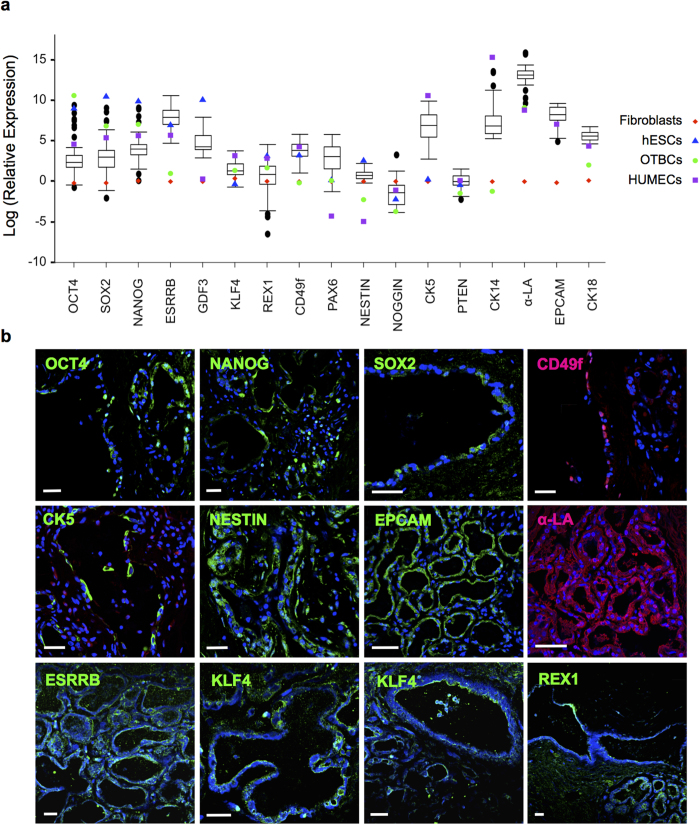
(**a**) Distributions of gene expression (RQ) amongst breastmilk cells (•) and reference cell line values [fibroblasts (♦), human embryonic stem cells (hESCs, Δ), OCT4 transfected breast cells (OTBCs, •) and human mammary epithelial cells (HUMECs, ■)]. Box plots represent breastmilk cell distributions where tails show the minimum and maximum values (excluding outliers) and upper and lower interquartile ranges; middle line represents the median. (**b**) Stained lactating breast tissue sections for OCT4, NANOG, SOX2, CD49f, CK5, NESTIN, EPCAM, α-LA, ESRRB, KLF4 and REX1.

Gene expression was tested both at the mRNA (qRT-PCR, Fig. 1a) and the protein levels (immunostaining of human lactating mammary tissues, Fig. 1b, Supplementary Figure 1). Breastmilk cell gene expression varied widely amongst participants (Fig. 1a, Table 2) where up to 10^5^ fold ranges were found within single genes. The genes ESRRB, CK5, CK14 and α-LA showed the highest relative expression, whilst REX1, NOGGIN and PTEN had the lowest expression (Table 2). Normal distributions were found for ESRRB, KLF4, CK5, CD49f, PAX6, NESTIN, NOGGIN, PTEN and CK18. Normality of gene expression distribution was indeterminate of α-LA (p = 0.052).

The core pluripotency transcription factors OCT4, SOX2 and NANOG had similar expression profiles (Fig. 1a), with the majority of breastmilk cells expressing these genes at high levels and in some cases comparable or even higher than in hESCs (Fig. 1a, Tables 2 and 3). It was found that 96% of the breastmilk cell samples expressed these genes at levels higher than fibroblasts, 2% had higher OCT4 expression than hESCs, and over 10% of the samples expressed the three genes at levels higher than HUMECs. All breastmilk samples had significantly higher ESRRB expression than fibroblasts, with a mean of greater than 6,400 times higher expression (p < 0.001) (Tables 2 and 3). Similarly, approximately 80% and 90% of the breastmilk cell samples had higher ESRRB expression than hESCs and HUMECs, respectively. GDF3 was expressed significantly higher in all breastmilk cell samples compared to both fibroblasts and resting human mammary epithelial cells (HUMECs) (p < 0.001 and p = 0.001, respectively) (Tables 2 and 3); however, all breastmilk cell samples had significantly lower GDF3 expression compared to hESCs (p < 0.001). For all cell lines tested, KLF4 and REX1 expression was within the range seen in breastmilk cells (Tables 2 and 3).

Approximately 35% of breastmilk cell samples expressed CD49f at levels up to 3 times higher than HUMECs, 70% of the samples at levels up to 12-fold higher than hESCs, and all samples were significantly higher than fibroblasts (p = 0.001) (Table 3). PAX6 was expressed in 90% of breastmilk samples up to 350 times more highly than both fibroblasts and hESCs (Fig. 1a). All breastmilk samples were also significantly higher than HUMECs for PAX6 and NESTIN (p < 0.001). NESTIN was significantly lower (up to 4 times) in breastmilk cells compared to hESCs (p = 0.002), and higher than fibroblasts for 90% of the samples. Gene expression for NOGGIN and PTEN for all the cell lines tested was within the range of breastmilk cells (Tables 2 and 3).

Mammary stem cell gene CK5 was variably expressed amongst the breastmilk cell samples, with HUMECs showing similar expression (Fig. 1a). On the other hand, myoepithelial marker CK14 was significantly lower in breastmilk cells, on average 70 times lower compared to HUMECs (p = 0.001). Expression of lactocyte markers α-LA, EPCAM and CK18 in breastmilk cells was up to 1000, 16 and 100 times higher, respectively than in HUMECs for the majority of samples tested (Table 3).

### Breastmilk gene expression correlates with genes of similar function

**Figure 2:**
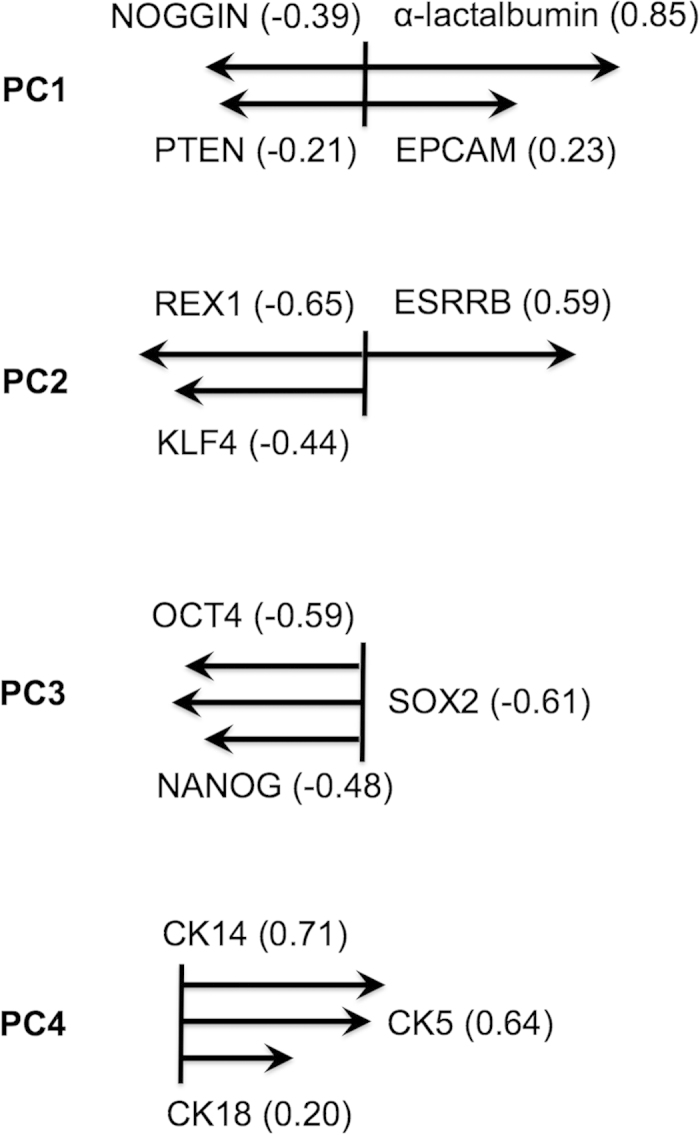
Graphical representation of gene associations found amongst breastmilk samples using sparse principle components.

Using principal component analysis (PCA), interactions for most genes tested were found to be within the first four components explaining 85.1% of the total variance within our gene expression profiles (Fig. 2). The first principal component (PC1) explained 52.6% of the variation in gene expression. Largely weighted α-LA and EPCAM clustered together and were in opposition to similarly weighted genes PTEN and NOGGIN. Within the second principal component (PC2) (Fig. 2), which explained a further 20.1% of the variation, the variance for ESRRB was opposite to the genes KLF4 and REX1. In the third principal component (PC3) (Fig. 2), which explained 6.5% of variation, OCT4, SOX2 and NANOG clustered together with similar weighting. In the forth and final principal component (PC4), which explained 5.9% of the variation, CK14 and CK5 clustered together with strong loadings with CK18, which had a weaker loading by comparison (Fig. 2).

### Breastmilk cell gene expression is associated with mother/infant demographic characteristics

**Figure 3:**
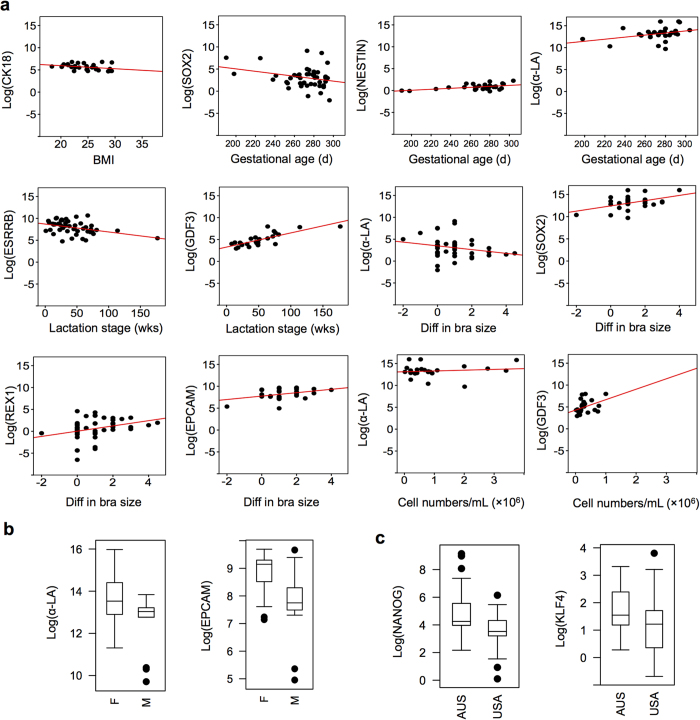
Scatterplots and boxplots for significantly associated linear and dichotomous characteristics and specific genes.

Higher maternal BMI was associated with lower expression of CK18 (p = 0.029). Higher gestational age at delivery (closer to term birth) was associated with higher α-LA (p = 0.040), higher NESTIN (p = 0.020) and lower SOX2 (p = 0.031) expression (Fig. 3). Early in lactation, ESRRB was more highly expressed (p = 0.005), whereas GDF3 was lower (p < 0.001). Larger changes in bra cup size between pre- and post-pregnancy were associated with lower SOX2 expression (p = 0.036) and higher REX1 (p = 0.047), α-LA (p = 0.022) and EPCAM (p = 0.047) expression (Fig. 3).

Cell content was positively associated with expression of α-LA (p = 0.027) and GDF3 (p = 0.046) Moreover, α-LA (p = 0.025) and EPCAM (p = 0.046) were higher in breastmilk samples of male infants (Fig. 3). A difference in gene expression of KLF4 (p = 0.010) and NANOG (p = 0.005) was found between Australian and USA samples (Fig. 3). No associations with demographic variables were identified for parity, maternal age or mode of delivery. When testing gene clusters and demographics, we found cell content to be significantly associated with the gene cluster PC1 (α-LA, EPCAM, PTEN and NOGGIN) (p < 0.001).

## Discussion

Breastmilk cells are emerging as a valuable non-invasive tool to examine the normal function, development and pathologies of the mammary gland. In this study, we examined breastmilk cellular heterogeneity at the molecular level, how it varies amongst lactating women, and how mother and infant characteristics may influence this variability. Importantly, we identified genes such as estrogen-related receptor-β (ESRRB), cytokeratin 5 (CK5), cytokeratin 14 (CK14) and α-lactalbumin (α-LA), which was universally expressed at high levels amongst the breastmilk cell samples examined. Moreover, genes regulating similar functions were found to have similar expression profiles, including pluripotency-regulating genes and lactation-associated genes. Mother-infant dyad characteristics showed significant correlations with specific genes, illustrating the high variability in gene expression amongst and within mothers and providing the basis for standardisation of studies that investigate the cellular content of breastmilk.

Expression profiles for most tested genes varied widely amongst breastmilk samples. These differences provide further evidence that breastmilk cell composition is highly variable amongst women and support the notion that breastmilk cells change in response to maternal and infant characteristics and are highly dependent on the specific mother-infant dyad^11^. The high expression recorded for the genes CK5, CK14 and CK18 is not surprising as these genes are representative of mammary stem/progenitor cells, myoepithelial cells and lactocytes respectively, all of which have been previously found in breastmilk^1^,^11^,^23^,^25^,^29^,^30^. In addition, these genes clustered closely together suggesting tight molecular regulation and/or involvement in similar cellular pathways in the lactating mammary gland. As expected, the milk protein α-LA and the pan-epithelial marker EPCAM showed higher expression in breastmilk cells compared to resting mammary gland cells (HUMECs), and the protein for both genes was prevalent in stained lactating breast tissues (Table 3). Our data therefore illustrate a strong epithelial signature and the presence of an epithelial hierarchy in breastmilk samples obtained from healthy mother-infant dyads, which is in agreement with previous literature^11^,^12^,^25^.

Interestingly, high expression of the gene estrogen-related receptor β (ESRRB) was observed in the majority of the breastmilk samples analysed and tended to be higher in breastmilk cells (range in breastmilk cells of 115–42,655 times higher than fibroblasts, whereas HUMECs had 2.73 times higher expression than fibroblasts) compared with HUMECs. ESRRB is an orphan nuclear receptor that displays disparate functions^31^. In *Drosophila* it functions as a metabolic switch during development^32^, however in murine embryonic stem cells it binds to NANOG to sustain pluripotency and cell self-renewal^33^. Its presence and significance has not yet been confirmed in hESCs, though it has been suggested to play a role in trophoblast stem cell differentiation^33^,^34^,^35^. ESRRB may be integral to mammary stem cell regulation and could potentially be regulated by fluctuating oestrogen that occurs naturally throughout pregnancy and lactation in the breast^36^. In the mammary gland, ESRRB has been shown to be co-expressed and correlated with estrogen receptor β (ER-β) in breast cancer biopsies; however, the significance of these observations is not well understood^37^. To our knowledge, this is the first report of ESRRB expression in breastmilk cells. The relatively high expression of this gene in breastmilk in comparison to resting mammary cells or human embryonic stem cells suggests that this receptor and its associated ligand(s) may play an important role in the human lactating mammary gland, potentially facilitating milk synthesis and mammary gland function. ESRRB functions may relate to stem/progenitor cell maturation and maintenance during lactation to support milk synthesis, a hypothesis that is supported by its close interaction with the genes KLF4 and REX1, both of which are known to participate in the control of multi-lineage differentiation and pluripotency in human embryonic stem cells^33^,^34^,^38^.

Other genes that were examined in this study that have not been previously considered in breastmilk cells are NOGGIN and PTEN. NOGGIN has been confirmed to be an important gene controlling embryonic development in the human^39^, and is also known as a bone morphogenic protein (BMP) antagonist, functioning to prevent differentiation of mammary epithelial cells^39^,^40^. In breastmilk cells, NOGGIN was not highly expressed (Fig. 1a), and is consistent with the high prevalence of differentiated lactocytes both in the lactating mammary gland and in breastmilk^11^,^41^,^42^. On the other hand, PTEN is a tumour suppressor influencing diverse functions such as cell cycle, apoptosis and metastasis^43^,^44^,^45^,^46^, known to be expressed in normal mammary tissue, with mutations and/or downregulation being characteristic of breast and other ectodermal cancers^47^,^48^. PTEN was minimally expressed in all breastmilk samples tested. Expression levels reported here, represent the normal expression in the lactating mammary gland and breastmilk of healthy women who do not have a history of breast cancer, and potentially reflect the rapid cycling of cells in the lactating epithelium. These results suggest that it would be of value to investigate expression of PTEN as a potential diagnostic biomarker in breastmilk of women that had previously had breast cancer or a family history for this disease. Both PTEN and NOGGIN were found to cluster together, but were inversely associated with the genes α-LA and EPCAM, suggesting potential opposing regulation and/or function of these gene pairs.

The pluripotency genes OCT4, SOX2 and NANOG were strongly correlated (Fig. 2). These genes have been shown to be the core transcription factors controlling multi-lineage differentiation and pluripotency of ESCs as well as induced pluripotent stem cells (iPSCs)^49^. Whilst not all breastmilk samples expressed high levels of these genes, some samples had similar levels of expression to hESCs, which is in accordance with previous literature^25^. The presence of these genes in the lactating mammary gland and in breastmilk at levels higher than the resting gland confirms previous reports from our and other laboratories^6^,^11^,^25^,^50^ and is also depicted in stained lactating breast tissue sections (Fig. 1b). The demonstration of a tight association of these genes further supports an important function in lactation and in the multi-lineage differentiation capability of breastmilk cells^25^. Other genes found to be expressed in breastmilk cells that may also regulate their differentiation capability are CD49f and PAX6, which are representative of mammary stem cells and the neuroectodermal lineage respectively^51^,^52^. Both genes had similar expression profiles in the breastmilk samples examined; however, PAX6 expression in breastmilk cells was much higher compared to resting breast cells, showing a significant upregulation during lactation. This signifies a function of PAX6 in the mammary gland during its remodelling associated with pregnancy and/or lactation. In addition, it may explain the known propensity of breastmilk stem cells to differentiate towards the neural lineage^25^,^27^. NESTIN is another known multi-lineage stem cell marker, which has been previously detected in breastmilk cells^23^. NESTIN expression was higher in breastmilk cells compared with HUMECs, which is indicative of an upregulation of the mammary stem/progenitor cell characteristics during lactation. NESTIN protein was also confirmed to be present in abundance in lactating breast tissues (Fig. 1b).

Importantly, large variation was found in gene expression amongst breastmilk samples, some of which can be explained by maternal and infant characteristics. Gestational age at delivery was associated with gene expression, in particular higher α-LA and NESTIN were found for infants with greater gestational age at delivery. This suggests that the mammary gland is more mature in mothers of term infants, containing more milk-secretory cells, but also more cells with progenitor properties thus being able to maintain some level of plasticity. For mothers of preterm infants, the levels of the stem cell marker SOX2 were much higher, accompanying the lower expression of α-LA and NESTIN compared to mothers of term infants. These findings suggest that the breast has not yet fully matured in women giving birth prematurely, providing a biological explanation for the observation that mothers of preterm infants often have a compromised initiation of lactation^53^. In this connection, a role in mammary development early in lactation may be exerted by ESRRB, which was more highly expressed earlier in lactation. The observed depletion of ESRRB+ cell populations, which occurs the longer a woman breastfeeds, may provide a an explanation for the known protection of breastfeeding against breast cancer^54^. On the other hand, GDF3 had a positive association with the stage of lactation, suggesting that this gene may be representative of a progenitor-like cell that is enriched towards later stages of lactation. This is in agreement with previous studies showing that during involution certain types of progenitor cells remain in the mammary gland and are thought to fuel the remodeling of the gland during the next pregnancy^55^. Both ESRRB and GDF3 may play fundamental roles in mammary development during pregnancy and lactation and are important to consider in further investigations.

A negative association was found between BMI and expression of CK18, a marker for luminal epithelial cells including lactocytes^30^. This suggests that women with a larger body mass index have less epithelial tissue capable of synthesizing milk. Previous studies have observed that maternal obesity before conception leads to a number of complications in postnatal breastfeeding including delayed onset of lactogenesis, impairment of lactogenesis II, and thus lower rates of breastfeeding initiation and shorter breastfeeding duration^56^,^57^,^58^,^59^. Our finding linking high maternal BMI with lower CK18 expression in breastmilk cells may explain the observed difficulties among breastfeeding mothers who are obese; this finding also provides a potential target for future clinical intervention.

Significant differences in the change of bra size between the pre-pregnancy and the postpartum mammary gland were found and were linked to higher levels of α-LA and EPCAM. This suggests that whilst prior studies have found that larger bra size does not necessarily translate to presence of more lactocytes, greater change in bra size, i.e. in breast volume^28^, is reflective of more epithelial cells, potentially resulting in higher milk production. This finding is interesting in light of Powe *et al.*, who reported that larger changes in bra size were linked with greater milk energy content; however, this association became non-significant after considering other factors^10^. The lower levels of SOX2 and the greater levels of REX1 expression observed with a greater change in maternal bra cup size may facilitate this process (Fig. 3).

In addition to demographic characteristics of the mother-infant dyad, breastmilk characteristics such as the cell content were found to be associated with gene expression. Higher breastmilk cell content was related to higher levels of α-LA and GDF3, suggesting the presence of more lactocytes and GDF3+ cells in cell-rich breastmilk, which typically reflects recent emptying of the breasts^6^. Breastmilk cell content was also significantly positively related to the gene cluster α-LA, EPCAM, PTEN and NOGGIN, which mainly represents epithelial cells. Moreover, infant sex was found to relate to epithelial/lactocyte genes such as α-LA and EPCAM, which were higher in the breastmilk from mothers of female infants. This suggests that these mothers had more lactocytes in their gland, which could potentially be associated with greater milk yield. Although this was not measured in the present study, it has been previously reported in captive rhesus macaques and in the dairy cow that mothers of female infants have greater milk yield compared to mothers of male infants^60^,^61^. This is in agreement with our findings, which may provide a mechanistic explanation of the fetus-driven differential remodeling of the mammary gland resulting in greater milk synthesis in mothers of female offspring.

Differences in breastmilk cell gene expression were observed between samples collected in Australia and the USA, although we are cautious in interpreting these results as other factors associated with different laboratory conditions or maternal milk supply may contribute to these observations. A further limitation of this study was the analysis of a pre-selected set of genes in all the cellular content of breastmilk. Therefore, some of the variation seen in gene expression may reflect the activity of a subgroup of cells, such as lactocytes or myoepithelial cells, whereas other variation may reflect the proportion of each cell type. Future studies will further delineate these questions by isolating and examining specific subpopulations of breastmilk cells as well as utilizing more comprehensive gene expression analyses, such as next generation sequencing. Finally, given that this study examined gene expression and effects of demographic traits in a cross-sectional dataset, it will be important to confirm and further examine these associations longitudinally.

## Conclusions

This study highlights the heterogeneity of breastmilk cell content and associations with characteristics of the breastfeeding mother-infant dyad. Interestingly, the lack of certain correlations, such as between breastmilk cell gene expression and maternal age, parity or mode of delivery, suggests that these characteristics may not important factors influencing total breastmilk cell gene expression in our cohorts. We identified core genes such as CK5, CK14, ESRRB and α-LA that are prevalent in breastmilk cells across mothers. Many of them show important interactions and may be involved in mammary gland function, lactation performance, and/or infant development in light of the recently reported integration of breastmilk cells into the infant^13^. Interestingly, some of the interactions found here between dyad demographic characteristics and genes such as α-LA and GDF3 may potentially explain abnormalities associated with low breastmilk supply and preterm birth, and warrant further investigation. In the future, breastmilk cellular analyses both at the protein and mRNA levels, considered together with dyad demographic characteristics, may be a useful routine practice in hospitals particularly in neonatal intensive care units, in the management of low milk supply, and during treatment of maternal and/or infant infections.

## Materials and Methods

### Breastmilk Sample Collection

The study was approved by the Human Research Ethics Committee of the University of Western Australia and the institutional review board (IRB) of The University of North Carolina at Chapel Hill, USA. All the methods were carried out in accordance with the approved guidelines. Breastfeeding dyads (*n* = 66) were recruited in Australia (*n* = 38) and the USA (*n* = 28), who were breastfeeding at least once daily (not necessarily exclusively) to attend a single session at either The University of Western Australia or the University of North Carolina. Exclusion criteria of the study were applied to participants that reported signs of breast, other organ or general/systemic infection, were pregnant or did not provide a sufficient sample to perform PCR with the extracted cellular RNA. All participants provided informed written consent and completed a confidential questionnaire including relevant demographic data. During this session, participants expressed a breastmilk sample (5–250 mL) under aseptic conditions with a Medela Symphony breast pump (Medela AG, Baar, Switzerland). Samples were transported to the laboratory immediately shielded from the light. Cell content and viability were measured, and RNA was extracted for RT-PCR analysis as previously described^25^.

### Breastmilk Cell Isolation

Breastmilk was diluted with equal volume of sterile phosphate buffered saline (PBS; pH 7.4, Gibco, Grand Island, NY) and centrifuged at 800 *g* for 20 minutes at 20 °C. The lipid layer and skim milk were removed, and the cell pellet was washed twice in PBS at 400 *g* for 5 minutes and was resuspended in PBS. The total cell content and viability of each sample were determined with a Neubauer haemocytometer by Trypan Blue exclusion. The cell pellet was stored at −80 °C until RNA extraction.

### Cell Culture

Cell lines used as positive and negative controls included adult dermal fibroblasts (Lonza, Walkersville, USA) and primary neonatal fibroblasts^25^, the Mel-2 embryonic stem cell line (StemCore, Brisbane, Australia), the OCT4-transduced breast cell (OTBC) line^62^, and a resting human mammary epithelial cell line (HUMEC) derived from normal resting breast tissue mammoplasties^25^. Cell lines were cultured in T75 flasks for several passages in a Sanyo CO_2_ incubator MCO-17AIC (Quantum Scientific, Queensland, Australia) held at a constant temperature of 37 °C at 5% CO_2_. Fibroblasts were cultured in DMEM/F12 supplemented with 20% fetal bovine serum (FBS; Fisher Biotec, Western Australia, Australia) and 1% antibiotic/antimycotic (Invitrogen, Victoria, Australia). Mel-2 cells were cultured in flasks coated with Matrigel (*In Vitro*, Victoria, Australia) at a density of 1.3 μL/cm^2^ in conditioned media from human fetal fibroblast feeder cells (CMKSR; conditioned mTeSR Stem Cell Technologies, Victoria, Australia) purchased from StemCore (Brisbane, Australia). Both OTBCs and HUMECs were cultured in HUMEC complete medium (Invitrogen, Victoria, Australia) supplemented with 1% antibiotic/antimycotic.

### RNA Extraction

Total RNA was extracted with the mini RNeasy extraction kit (Qiagen, Valencia, CA). Cell pellets were incubated in 600 μL of RLT buffer for 10 minutes and transferred to a separate Eppendorf tube whereby they were triturated through a 21G needle syringe 10 times. Lysate was mixed well and equal volumes of 70% ethanol was added before mixing and spinning the lysate through the provided spin column at a maximum speed of 8,000 *g* for 30 seconds. Flow through was discarded and 700 μL of RW1 solution was added to the spin column before spinning at 8,000 *g* for 30 seconds. Following this, 500 μL of RPE buffer were added and spun at 8,000 *g* for 30 seconds. This was repeated after the flow-through was discarded, and it was spun for 2 minutes at 8,000 *g*. The spin column was placed in a new tube and 30 μL of RNAse free water was added to the center of the column, incubated for 10 minutes on ice before spinning for a final time at 8,000 *g* for 1 minute. After the RNA was eluted, RNA quantitation and purity was measured using a Nanodrop 1000. RNA obtained was of an acceptable quality fitting in the absorbance at 260/280 ratio of 1.8–2.2.

### cDNA Generation

Total RNA was reverse transcribed using the high-capacity cDNA archive kit (Applied Biosystems, Carlsbad, CA). A 50-μL reaction was created by adding prescribed volumes of each component contained within the kit, to make up the cDNA master mix to 25 μL of the RNA diluted in Ultrapure RNAse free water (Gibco). Samples were incubated in a Bio-Rad C1000 96 well gradient block thermo cycler and held at 25 °C for 10 minutes, 37 °C for 120 minutes, 85 °C for 5 minutes, and held at 4 °C until collected. cDNA was stored at −20 °C until required for quantitative real-time polymerase chain reaction (qRT-PCR).

### Quantitative Real-Time Polymerase Chain Reaction (qRT-PCR)

Gene transcription was quantified by qRT-PCR using hydrolytic probes (Taqman, Applied Biosystems; Supporting Information Table S1) with the 7500 Fast RT-PCR system (Applied Biosystems). Each sample was measured in triplicate or in few cases in duplicate when the extracted RNA was not adequate. Genes were standardized to fibroblasts, and each sample was controlled with an in house regulator GAPDH. Fold change in gene expression for each sample and experimental condition was calculated as 2^Ct(control)–Ct(sample)^ ± SD and relative quantitation was determined for each replicate. Repeated measures of the samples were averaged and the standard deviations were calculated. Standard deviations were used for quality control of the data and means were used for statistical analyses.

### Immunostaining of mammary tissues

Human biopsied mammary tissues from lactating women were obtained from the tissue archive of the School of Anatomy, Physiology and Human Biology, The University of Western Australia. Formalin-fixed and paraffin-embedded tissues were sectioned at 5 μm thickness and immunostained for fluorescence microscopy as described in Hassiotou *et al.* 2012^25^. The primary and secondary antibodies used are shown in Suppl. Table 2.

### Flow cytometry

Flow cytometric analysis was conducted in cells isolated from freshly expressed breastmilk collected from two breastfeeding women according to Hassiotou *et al.* 2012^25^. In short, isolated cells were incubated with fixative (1.5% Paraformaldehyde: BDH, Poole, England; 1% Sucrose: Merck, Darmstadt, Germany in phosphate buffered saline/PBS: Invitrogen, Mulgrave, Victoria) for 10 minutes followed by washing in PBS and were then centrifuged for 30 seconds. Permeabilisation occurred by adding Tween 20 (USB, Cleveland, USA) 0.05% for 10 minutes to the cells. Primary antibodies (Table S2) were added to the cells in Tween 20 0.05% with 2% fetal bovine serum (Borogen, Essendon, Australia) and incubated for 1 hour in the dark at 4 °C following washing. Cells were washed twice and incubated with 1:200 secondary antibody solution (Table S2) as above for 30 minutes in the dark at 4 °C. Cells were washed twice in PBS/Tween 0.05%, and fixative was added. Appropriate negative internal controls were also prepared (no primary antibody). Data acquisition was done with a FACS Calibur Flow Cytometer (Becton Dickinson, Franklin Lakes, NJ, http://www.bd.com) and data analysis was done with FlowJo.

### Statistical Analyses

Statistical analyses were carried out using R 2.9.0 for Mac OSX, with additional packages PMA^63^ and MASS^64^ for sparse principle component analysis and robust regression, respectively. Summary demographics and sample details are presented as means (ranges) in the results section and medians and ranges in Table 1. Demographic data were coded as follows: bra cup sizes as A = 1, B = 2, C = 3, D = 4, DD = 5, E = 6, F = 7, FF = 8, G = 9, H = 10; mode of delivery as either vaginal delivery (including assisted) or caesarean section (emergency and elective). Gene expression was determined by relative quantitation (RQ) compared to the control fibroblasts. RQ data were generally logged due to the order of magnitude changes that exist between samples. Missing data due to varying levels of RNA extracted from breastmilk samples were taken into consideration using statistical analyses as described below. Multiple models were used to account for non-uniformity of the data, as explained below. In general, p-values below 0.05 were considered statistically significant, unless stipulated otherwise. Very small p-values are reported as p < 0.001. No adjustments were made for multiple comparisons other than the selection of more stringent alpha values for z-score analysis. Borderline associations (0.05 ≤ p < 0.1) have not been reported.

To examine how gene expression varied between breastmilk cells, gene expression distributions were examined graphically and tested for normality. Normality was tested for each gene by the Shapiro-Wilk normality test. Data were assumed to be normally distributed when p ≥ 0.1, indeterminate when 0.05 ≤ p < 0.1 and not normally distributed when p < 0.05. To determine whether cell line values fitted within the distribution of breastmilk cells, z-scores and associated p-values were calculated based on whether cell line values sat outside of 99% of the distribution. As such, any z-scores for cell lines that sat outside of |2.56| were reported to be significantly different from breastmilk cell values (p < 0.01). The hESC cell line was not tested for the mammary differentiation genes (α-LA, EPCAM, CK14 or CK18) and OTBCs were not tested for EPCAM, CK5 or GDF3 as they were not considered relevant controls of these genes.

Possible gene-gene interactions were determined using sparse principle component analysis (PCA’s) that examined logged RQ variance to determine different components. The method developed by Witten *et al.* 2009, used here, allows for calculations of principle components in the presence of missing data. Using the defaults: non-orthoganality, 20 reiterations and centered data^63^, we looked at the first four principle components with a sum of the absolute values equal to 2. These parameters were set based on four perceived groupings (pluripotency genes, stem cell genes, mammary genes and a tumour suppressor gene). Another principle component was added to examine if there might be further groups than this; however, it was dropped out because the explanation of the variance between the 4^th^ and 5^th^ component did not increase by more than 5% when using a greater group number. Loadings of >|0.1| only were considered.

Univariate relationships between measured gene expression (log[RQ]) and continuous demographic data (maternal BMI, maternal age, difference in bra size, parity, current infant age, gestational age at delivery, and sample cell content) were assessed using regression. For all genes, ordinary least squares (OLS) regression models were fit for each demographic, after centering to meaningful theoretical values for each demographic (BMI on 25; Gestational age at delivery of 280 days [40 weeks]) and logging RQ values. Appropriateness of the model was assessed using graphical residual analysis. Where residuals were found not to be normally distributed, robust linear modeling was applied^65^ and considered both the Huber and Tukey bisquared residual corrections. T-value output was converted to p-values for interpretation. Ordinary least squares regression was used for the gene ESRRB against the demographic lactation stage, and for GDF3 compared to cell content. Genes CK18, NESTIN and REX1 were found to be more appropriately represented applying the Huber correction and the exception of cell content and α-LA where the Huber correction was also found to be more appropriate. The Tukey correction was suited for the genes α-LA, SOX2, EPCAN and GDF3 when examining correlations with lactation stage.

For dichotomous demographic data (primi versus multigravida, infant sex, mode of delivery), differences in gene expression between categories were tested with one or more of Student’s *t*-test, Welch’s *t*-test, or the Wilcoxon signed rank test as follows. Where the Shapiro-Wilk test indicated that the data were not normally distributed (p < 0.05), the Wilcoxon sign rank test was used. Where normality of the data was indicated (p ≥ 0.1), Bartlett’s test was used to determine whether the assumption of equal variances held. Where this was considered to be the case (p ≥ 0.1) the Student’s *t*-test was used, and where it was not (p < 0.05) Welch’s *t*-test was used. In situations where either the Shapiro-Wilk or Bartlett’s test was ambiguous (0.05 ≤ p < 0.1) both conditions were run. When examining the difference between infant sexes for different genes, the multi-sex infant pair was excluded. The Welch test was found to be the most appropriate analysis for the gene α-LA and Wilcoxon for the genes EPCAM, KLF4 and NANOG. Associations between the discovered groups of genes with individual demographics were determined using the values generated by the sparse principle components. Ordinary least squares regression was then applied to determine associations with continuous demographic data.

## Additional Information

**How to cite this article**: Twigger, A.-J. *et al.* Gene expression in breastmilk cells is associated with maternal and infant characteristics. *Sci. Rep.* **5**, 12933; doi: 10.1038/srep12933 (2015).

## Supplementary Material

Supplementary Information

## Figures and Tables

**Table 1:** Demographic and breastmilk sample characteristics of the mother/infant dyads (N = 66).

	Median	Range
**Maternal characteristics**		
Age (years)	34	21–44
Body mass index (BMI)	23.2	17.5–37.4
Change in Bra size (cup sizes)*	C to D	A–H
Parity	1	1–4
**Infant characteristics**		
Age (weeks)	30.5	1–177
Infant gestational age (weeks)	39.3	27.1–43.4
**Breastmilk samples**		
Cell viability (%)	97.9	85.3–100
Volume of breastmilk provided (mL)	55	6–240
Total breastmilk cell count (×10^6^)	16.4	1.6–201.3
Breastmilk cell content (cells/mL milk, ×10^5^)	2.77	0.17–37.5

^*^Cup sizes represent Australian bra sizes.

**Table 2:** Range of gene expression in breastmilk cells, where N represents the total number of samples tested per gene.

Gene	N	Mean	Median	STDEV	Range
OCT4	48	512	10.73	2,004	0.47–12,924
SOX2	46	438	20.61	1,589	0.13–9,190
NANOG	47	548	56.14	1,815	1.11–9,399
ESRRB	49	6,488	2,880	8,828	115–42,656
GDF3	25	492	74.39	8.23	18.71–2,919
KLF4	50	6.88	3.73	19.56	0.50–44.85
REX1	44	10.20	2.42	895	0.001–98.79
CD49f	29	83.00	47.43	94.12	2.87–351
PAX6	29	51.04	21.92	74.25	0.28–346
NESTIN	29	2.80	2.13	2.04	0.83–9.43
NOGGIN	29	1.49	9.25	5.02	0.02–27.17
CK5	29	2,761	1,057	4,424	16.11–21,621
PTEN	29	1.38	1.00	1.07	0.11–4.72
CK14	29	66,305	987	206,174	204–857,379
α-LA*	29	1,379,623	548,334	2,390,070	16,451–8,672,119
EPCAM	29	6,135	4,005	5,068	142–16,180
CK18	29	352.0	283	241	107–900

The means, medians, STDEV, and ranges represent the relative expression of each gene compared to the control cell line (fibroblasts).

^*^α-LA: alpha-lactalbumin.

**Table 3:** Comparison of gene expression in breastmilk cells with negative and positive cell lines for the examined genes (fibroblasts, ESCs, and HUMECs).

Gene	RQ of milk cellsMean ± STDEV	RQ OTBCs	RQ HUMECs	RQ hESCs	P-valueFibroblasts	P-valueOTBCs	P-valueHUMECs	P-valuehESCs
OCT4	512 ± 2,004	41,216 ± 530	102 ± 17.90	8,282 ± 1,668	0.082	<0.001	0.215	0.003
SOX2	438 ± 1,589	959 ± 39.67	224 ± 22.59	36,029 ± 6,061	0.074	0.046	0.152	<0.001
NANOG	548 ± 1,815	1,182 ± 26.50	291 ± 22.69	20,028 ± 3,452	0.011	0.053	0.203	<0.001
ESRRB	6,488 ± 8,828	2.73 ± 1.73	305 ± 48.01	1,070 ± 95.01	<0.001	<0.001	0.063	0.251
GDF3	492 ± 8.23	—	1.37 ± 0.94	24,171 ± 1,041	<0.001	—	0.001	<0.001
KLF4	6.88 ± 19.56	3.96 ± 0.42	24.52 ± 3.58	0.72 ± 0.06	0.152	0.480	0.040	0.041
REX1	10.20 ± 895	5.44 ± 1.08	17.71 ± 4.01	23.23 ± 7.42	0.401	0.330	0.176	0.149
CD49f	83.00 ± 94.12	0.85 ± 0.19	72.85 ± 9.23	25.34 ± 3.13	<0.001	<0.001	0.348	0.316
PAX6	51.04 ± 74.25	1.13 ± 0.10	0.01 ± 0.01	1.18 ± 0.18	0.062	0.071	<0.001	0.075
NESTIN	2.80 ± 2.04	0.11 ± 0.01	0.01 ± 0.00	13.16 ± 2.21	0.095	<0.001	<0.001	0.002
NOGGIN	1.49 ± 5.02	0.02 ± 0.00	0.35 ± 0.01	0.11 ± 0.00	0.206	0.087	0.425	0.312
CK5	2,761 ± 4,424	—	40,834 ± 10,363	1.29 ± 0.11	<0.001	—	0.022	<0.001
PTEN	1.38 ± 1.07	0.23 ± 0.03	1.13 ± 0.26	0.65 ± 0.15	0.496	0.052	0.448	0.316
CK14	66,305 ± 206,174	0.30 ± 0.23	4,671,694 ± 155,982	—	<0.001	<0.001	<0.001	—
α-LA*	1,379,623 ± 2,390,070	9,347 ± 4,892	6,807 ± 2,155	—	<0.001	0.003	0.002	—
EPCAM	6,135 ± 5,068	—	1,201	—	<0.001	—	0.171	—
CK18	352 ± 241	7.73 ± 0.36	80.29 ± 8.47	—	<0.001	<0.001	0.030	—

P values were calculated from t-values generated from z-score analysis for the log of the relative expression (RQ) values.

## References

[b1] Hassiotou F. & Geddes D. Anatomy of the human mammary gland: Current status of knowledge. Clinical anatomy 26, 29–48, 10.1002/ca.22165 (2013). 22997014

[b2] Neville M. C. *et al.* Studies on human lactation. I. Within-feed and between-breast variation in selected components of human milk. The American journal of clinical nutrition 40, 635–646 (1984). 6475828 10.1093/ajcn/40.3.635

[b3] Khan S. *et al.* Investigation of short-term variations in term breast milk composition during repeated breast expression sessions. Journal of human lactation: official journal of International Lactation Consultant Association 29, 196–204, 10.1177/0890334412470213 (2013). 23422498

[b4] Qian J., Chen T., Lu W., Wu S. & Zhu J. Breast milk macro- and micronutrient composition in lactating mothers from suburban and urban Shanghai. Journal of paediatrics and child health 46, 115–120, 10.1111/j.1440-1754.2009.01648.x (2010). 20105254

[b5] Mitoulas L. R. *et al.* Variation in fat, lactose and protein in human milk over 24h and throughout the first year of lactation. British Journal of Nutrition 88, 29, 10.1079/bjn2002579 (2007). 12117425

[b6] Hassiotou F. *et al.* Breastmilk cell and fat contents respond similarly to removal of breastmilk by the infant. PloS one 8, e78232, 10.1371/journal.pone.0078232 (2013). 24223141 PMC3819380

[b7] Bauer J. & Gerss J. Longitudinal analysis of macronutrients and minerals in human milk produced by mothers of preterm infants. Clinical nutrition 30, 215–220, 10.1016/j.clnu.2010.08.003 (2011). 20801561

[b8] Molinari C. E., Casadio Y. S., Hartmann B. T., Arthur P. G. & Hartmann P. E. Longitudinal analysis of protein glycosylation and beta-casein phosphorylation in term and preterm human milk during the first 2 months of lactation. British Journal of Nutrition 110, 105–115, 10.1017/s0007114512004588 (2013). 23182305

[b9] Bachour P., Yafawi R., Jaber F., Choueiri E. & Abdel-Razzak Z. Effects of Smoking, Mother’s Age, Body Mass Index, and Parity Number on Lipid, Protein, and Secretory Immunoglobulin A Concentrations of Human Milk. Breastfeeding Medicine 7, 179–188, 10.1089/bfm.2011.0038 (2012). 22166069

[b10] Powe C. E., Knott C. D. & Conklin-Brittain N. Infant Sex Predicts Breast Milk Energy Content. Am. J. Hum. Biol. 22, 50–54, 10.1002/ajhb.20941 (2010). 19533619

[b11] Hassiotou F., Geddes D. T. & Hartmann P. E. Cells in human milk: state of the science. Journal of human lactation: official journal of International Lactation Consultant Association 29, 171–182, 10.1177/0890334413477242 (2013). 23515088

[b12] Hassiotou F. *et al.* Maternal and infant infections stimulate a rapid leukocyte response in breastmilk. Clinical & Translational Immunology 2, e3, 10.1038/cti.2013.1 (2013). 25505951 PMC4232055

[b13] Hassiotou F. *et al.* Breastmilk stem cells transfer from the mother to neonatal organs: a route of migration and integration. *Bi-annual Conference of International Society for Research in Human Milk and Lactation* (2014).

[b14] Jain L. *et al.* *In vivo* distribution of human milk leucocytes after ingestion by newborn baboons. Archives of Disease in Childhood 64, 930–933, 10.1136/adc.64.7_Spec_No.930 (1989). 2774634 PMC1590089

[b15] Brooker B. E. The epithelial cells and cell fragments in human milk. Cell and tissue research 210, 321–332 (1980). 7407874 10.1007/BF00237619

[b16] Thomas E., Zeps N., Rigby P. & Hartmann P. Reactive oxygen species initiate luminal but not basal cell death in cultured human mammary alveolar structures: a potential regulator of involution. Cell death & disease 2, e189, 10.1038/cddis.2011.69 (2011). 21814287 PMC3181416

[b17] Tiede B. & Kang Y. From milk to malignancy: the role of mammary stem cells in development, pregnancy and breast cancer. Cell research 21, 245–257, 10.1038/cr.2011.11 (2011). 21243011 PMC3193434

[b18] Buehring G. C. Culture of human mammary epithelial cells keeping abreast with a new method. J. Natl. Cancer Inst. 49, 1433-& (1972). 4568089

[b19] Gaffney E. V., Polanowski F. P., Blackburn S. E., Lambiase J. T. & Burke R. E. Cultures of normal human mammary cells. Cell Differentiation 5, 69–81 (1976). 971488 10.1016/0045-6039(76)90001-4

[b20] TaylorPapadimitriou J., Taylor Papadimitriou M., Shearer, M. G. P. & Stoker. Growth requirements of human mammary epithelial cells in culture. International journal of cancer 20, 903–908 (1977). 10.1002/ijc.2910200613 304048

[b21] Stoker M., Perryman M. & Eeles R. Clonal Analysis of Morphological Phenotype in Cultured Mammary Epithelial Cells from Human Milk. Proceedings of the Royal Society B: Biological Sciences 215, 231–240, 10.1098/rspb.1982.0039 (1982). 6127705

[b22] Russo J., Furmanski P., Bradley R., Wells P. & Rich M. A. Differentiation of normal human mammary epithelial cells in culture: an ultrastructural study. American Journal of Anatomy 145, 57–77 (1976). 129001 10.1002/aja.1001450105

[b23] Cregan M. D. *et al.* Identification of nestin-positive putative mammary stem cells in human breastmilk. Cell and tissue research 329, 129–136, 10.1007/s00441-007-0390-x (2007). 17440749

[b24] Fan Y., Chong Y. S., Choolani M. A., Cregan M. D. & Chan J. K. Unravelling the mystery of stem/progenitor cells in human breast milk. PloS one 5, e14421, 10.1371/journal.pone.0014421 (2010). 21203434 PMC3010984

[b25] Hassiotou F. *et al.* Breastmilk is a novel source of stem cells with multilineage differentiation potential. Stem cells 30, 2164–2174, 10.1002/stem.1188 (2012). 22865647 PMC3468727

[b26] Twigger A. J. *Characterization of human breastmilk stem cells cultured towards the neural lineage* Biomedical Science (Hons) thesis, The University of Western Australia, (2012).

[b27] Twigger A. J., Hodgetts S., Filgueira L., Hartmann P. E. & Hassiotou F. From breast milk to brains: the potential of stem cells in human milk. Journal of human lactation : official journal of International Lactation Consultant Association 29, 136–139, 10.1177/0890334413475528 (2013). 23515086

[b28] Cox D. B. The Morphological and Functional Development of the Human Breast During Pregnancy and Lactation Doctor of Philosophy thesis, The University of Western Australia, (1996).

[b29] Ahmad R. *et al.* Functional neuronal cells generated by human parthenogenetic stem cells. PloS one 7, e42800, 10.1371/journal.pone.0042800 (2012). 22880113 PMC3412801

[b30] Böcker W. *et al.* Common Adult Stem Cells in the Human Breast Give Rise to Glandular and Myoepithelial Cell Lineages: A New Cell Biological Concept. Laboratory Investigation 82, 737–746, 10.1097/01.lab.0000017371.72714.c5 (2002). 12065684

[b31] Pardo I. *et al.* Next-generation transcriptome sequencing of the premenopausal breast epithelium using specimens from a normal human breast tissue bank. Breast cancer research: BCR 16, R26, 10.1186/bcr3627 (2014). 24636070 PMC4053088

[b32] Tennessen J. M., Baker K. D., Lam G., Evans J. & Thummel C. S. The Drosophila estrogen-related receptor directs a metabolic switch that supports developmental growth. Cell Metabolism 13, 139–148, 10.1016/J.Cmet.2011.01.005 (2011). 21284981 PMC3072597

[b33] Festuccia N. *et al.* Esrrb is a direct Nanog target gene that can substitute for Nanog function in pluripotent cells. Cell stem cell 11, 477–490, 10.1016/j.stem.2012.08.002 (2012). 23040477 PMC3473361

[b34] Zhang X., Zhang J., Wang T., Esteban M. A. & Pei D. Esrrb activates Oct4 transcription and sustains self-renewal and pluripotency in embryonic stem cells. The Journal of biological chemistry 283, 35825–35833, 10.1074/jbc.M803481200 (2008). 18957414

[b35] Percharde M. *et al.* Ncoa3 functions as an essential Esrrb coactivator to sustain embryonic stem cell self-renewal and reprogramming. Genes & development 26, 2286–2298, 10.1101/gad.195545.112 (2012). 23019124 PMC3475801

[b36] Neville M. C., McFadden T. B. & Forsyth I. Hormonal Regulation of Mammary Differentiation and Milk Secretion. Journal of mammary gland biology and neoplasia 7, 49–66 (2002). 12160086 10.1023/a:1015770423167

[b37] Ariazi E. A., Clark G. M. & Mertz J. E. Estrogen-related receptor alpha and estrogen-related receptor gamma associate with unfavorable and favorable biomarkers, respectively, in human breast cancer. Cancer research 62, 6510–6518 (2002). 12438245

[b38] Feng B. *et al.* Reprogramming of fibroblasts into induced pluripotent stem cells with orphan nuclear receptor Esrrb. Nature cell biology 11, 197–203, 10.1038/ncb1827 (2009). 19136965

[b39] Pera M. F. *et al.* Regulation of human embryonic stem cell differentiation by BMP-2 and its antagonist noggin. Journal of cell science 117, 1269–1280, 10.1242/jcs.00970 (2004). 14996946

[b40] Guo D., Huang J. & Gong J. Bone morphogenetic protein 4 (BMP4) is required for migration and invasion of breast cancer. Molecular and cellular biochemistry 363, 179–190, 10.1007/s11010-011-1170-1 (2012). 22167620

[b41] Stingl J. *et al.* Purification and unique properties of mammary epithelial stem cells. Nature 439, 993–997, 10.1038/nature04496 (2006). 16395311

[b42] Visvader J. E. Keeping abreast of the mammary epithelial hierarchy and breast tumorigenesis. Genes & development 23, 2563–2577, 10.1101/gad.1849509 (2009). 19933147 PMC2779757

[b43] Tamura M. *et al.* Inhibition of Cell Migration, Spreading, and Focal Adhesions by Tumor Suppressor PTEN. Science 280, 1614–1617 (1998). 9616126 10.1126/science.280.5369.1614

[b44] Lu Y. *et al.* The PTEN/MMAC1/TEP tumour suppressor gene decreases cell growth and induces apoptosis and anoikis in breast cancer cells. Oncogene 18, 7034–7045, 10.1038/Sj.Onc.1203183 (1999). 10597304

[b45] Waite K. A. & Eng C. Protean PTEN: form and function. American journal of human genetics 70, 829–844, 10.1086/340026 (2002). 11875759 PMC379112

[b46] Weng L., Brown J. & Eng C. PTEN coordinates G1 arrest by down-regulating cyclin D1 via its protein phosphotase activity and up-regulating p27 via its lipid phosphotase activity in a breast cancer model. Human Molecular Genetics 10, 599–604 (2001). 11230179 10.1093/hmg/10.6.599

[b47] Zhang H. Y., Liang F., Jia Z. L., Song S. T. & Jiang Z. F. mutation, methylation and expression in breast cancer patients. Oncology letters 6, 161–168, 10.3892/ol.2013.1331 (2013). 23946797 PMC3742525

[b48] Li J. P. T. E. N., a Putative Protein Tyrosine Phosphatase Gene Mutated in Human Brain, Breast, and Prostate Cancer. Science 275, 1943–1947, 10.1126/science.275.5308.1943 (1997). 9072974

[b49] Takahashi K. *et al.* Induction of pluripotent stem cells from adult human fibroblasts by defined factors. Cell 131, 861–872, 10.1016/j.cell.2007.11.019 (2007). 18035408

[b50] Roy S. *et al.* Rare somatic cells from human breast tissue exhibit extensive lineage plasticity. PNAS 110, 4598–4603, 10.1073/pnas.1218682110 (2013). 23487770 PMC3607035

[b51] Visvader J. E. & Lindeman G. J. Mammary stem cells and mammopoiesis. Cancer research 66, 9798–9801, 10.1158/0008-5472.CAN-06-2254 (2006). 17047038

[b52] Kioussi C. *et al.* Pax6 is essential for establishing ventral-dorsal cell boundaries in pituitary gland development. Proceedings of the National Academy of Sciences of the United States of America 96, 14378–14382, 10.1073/pnas.96.25.14378 (1999). 10588713 PMC24444

[b53] Cregan M. D., De Mello T. R., Kershaw D., McDougall K. & Hartmann P. E. Initiation of lactation in women after preterm delivery. Acta Obstet Gynecol Scand 81, 870–877 (2002). 12225305 10.1034/j.1600-0412.2002.810913.x

[b54] Kotsopoulos J. *et al.* Breastfeeding and the risk of breast cancer in BRCA1 and BRCA2 mutation carriers. Breast cancer research: BCR 14, R42, 10.1186/bcr3138 (2012). 22405187 PMC3446376

[b55] Wagner K. *et al.* An adjunct mammary epithelial cell population in parous females: its role in functional adaptation and tissue renewal. Development 129, 1377–1386 (2002). 11880347 10.1242/dev.129.6.1377

[b56] Rasmussen K. M. Association of maternal obesity before conception with poor lactation performance. Annual review of nutrition 27, 103–121, 10.1146/annurev.nutr.27.061406.093738 (2007). 17341160

[b57] Rasmussen K. M., Hilson J. A. & Kjolhede C. L. Obesity may impair lactogenesis II. Journal of Nutrition 131, 3009S–3011S (2001). 11694637 10.1093/jn/131.11.3009S

[b58] Nommsen-Rivers L. A., Chantry C. J., Peerson J. M., Cohen R. J. & Dewey K. G. Delayed onset of lactogenesis among first-time mothers is related to maternal obesity and factors associated with ineffective breastfeeding. Am J Clin Nutr 92, 574–584, 10.3945/ajcn.2010.29192 (2010). 20573792

[b59] Amir L. H. & Donath S. A systematic review of maternal obesity and breastfeeding intention, initiation and duration. BMC pregnancy and childbirth 7, 9, 10.1186/1471-2393-7-9 (2007). 17608952 PMC1937008

[b60] Hinde K. Richer milk for sons but more milk for daughters: Sex-biased investment during lactation varies with maternal life history in rhesus macaques. American journal of human biology: the official journal of the Human Biology Council 21, 512–519, 10.1002/ajhb.20917 (2009). 19384860

[b61] Hinde K., Carpenter A. J., Clay J. S. & Bradford B. J. Holsteins favor heifers, not bulls: biased milk production programmed during pregnancy as a function of fetal sex. PloS one 9, e86169, 10.1371/journal.pone.0086169 (2014). 24498270 PMC3911898

[b62] Beltran A. S. *et al.* Generation of tumor-initiating cells by exogenous delivery of OCT4 transcription factor. Breast cancer research: BCR 13, R94, 10.1186/bcr3019 (2011). 21952072 PMC3262206

[b63] Witten D. M., Tibshirani R. & Hastie T. A penalized matrix decomposition, with applications to sparse principal components and canonical correlation analysis. Biostatistics 10, 515–534, 10.1093/biostatistics/kxp008 (2009). 19377034 PMC2697346

[b64] Venables W. N. & Ripley B. D. Modern Applied Statistics with S. Vol. Fourth (Springer, 2002).

[b65] Fox J. & Weisberg S. in An R and S-Plus companion to applied regression (ed Fox John) (Sage Publications, 2002).

