# Supplementary material for: Gene expression in breastmilk cells is associated with maternal and infant characteristics
Source: Sci Rep. 2015 Aug 10;5:12933. doi: 10.1038/srep12933 (PMC4542700; doi:10.1038/srep12933)
Supplement: Supplementary Information [file srep12933-s1.pdf]

SUPPLEMENTARY INFORMATION

Gene expression in breastmilk cells is associated with maternal and infant characteristics

Alecia-Jane Twigger[1], Anna R. Hepworth[1], Ching Tat Lai[1], Ellen Chetwynd[2], Alison M. Stuebe[2], Pilar Blancafort[3,4], Peter E. Hartmann[1], Donna T. Geddes[1], Foteini Kakulas[1]

Supplementary Figure 1. Flow cytometric analysis of 2 breastmilk cell samples collected from 2 breastfeeding women showing expression of ESRRB, KLF4 and REX1.

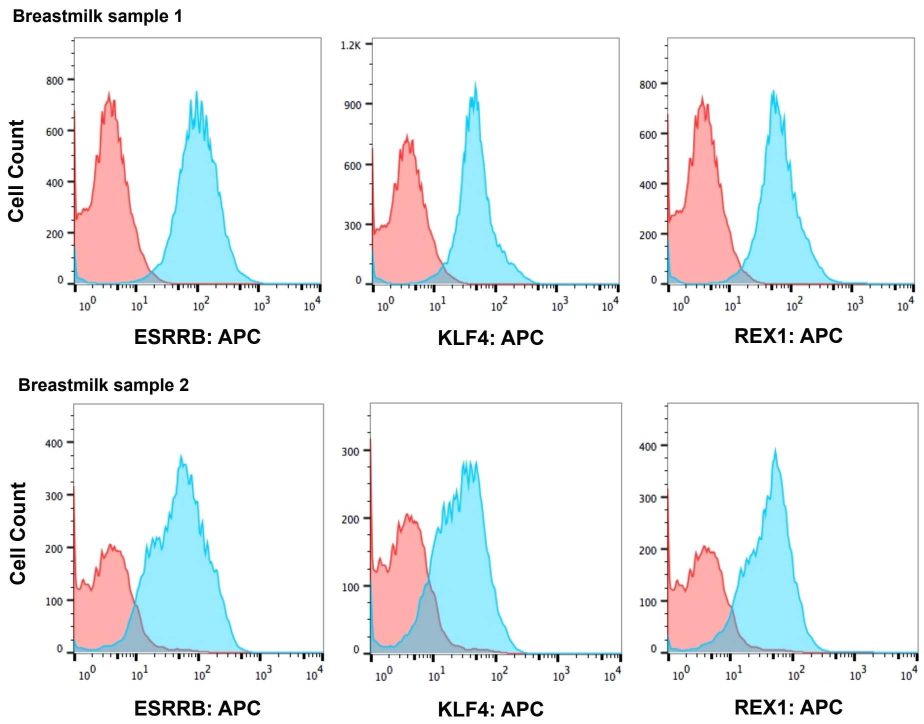

Supplementary Table 1. Taqman probes used for RT-PCR.

Gene	Applied Biosystems Reference Number
HuGAPDH	Hs03929097_g1
OCT4 (POU5F1)	Hs03005111_g1
SOX2	Hs01053049_s1
NANOG	Hs02387400_g1
ESRRB	Hs01584024_m1
GDF3	Hs00220998_m1
KLF4	Hs00358836_m1
REX1	Hs00810654_m1
CD49f	Hs01041011_m1
PAX6	Hs00240871_m1
NESTIN	Hs04187831_g1
NOGGIN	Hs00271352_s1
CK5	Hs00361185_m1
PTEN	Hs02621230_s1
CK14	Hs00265033_m1
α-LA	Hs00182028_m1
EPCAM	Hs00901885_m1
CK18	Hs02827483_g1

Supplementary Table 2. Antibodies used for immunostaining.

Antibody	Company	Catalogue number	Dilution for Immunostaining	Dilution for Flow Cytometry
OCT4 [illegible]	Miltenyi (Stemgent)	130-095-635	1:100	-
SOX2	Miltenyi (Stemgent)	130-095-636	1:50	-
NANOG	Abcam	ab80892	1:100	-
NANOG	Cell signalling	48935	-	-
CD49f	Peprotech/Serotec	SEMCA699BT	1:100	-
NESTIN	Miltenyi (Stemgent)	130-095-648	1:100	-
CK5	Leica (Novocastra)	CK5-CE-S	1:50	-
α-LA	Dako	A057901	1:500	-
EPCAM	Exbio	11-581-C100	1:300	-
ESRRB	Thermo Fisher	PA5-26070	1:50	1:50
KLF4	Abcam	ab72543	1:50	1:50
REX1	Abcam	ab72543	1:50	1:50
SSEA4	Stemgent	09-0006	-	-
AlexaFluor 488	Life Technologies	A21202	1:100	1:200
AlexaFluor 546	Life Technologies	A10036	-	1:200
DAPI	Roche	10236276001	1:100	-
